# Innovative hyaluronic acid-decorated quatsomes for enhanced renal targeting: design, optimization and *in-vivo* biochemical and nephroprotective assessments

**DOI:** 10.3389/jpps.2026.16787

**Published:** 2026-07-24

**Authors:** Sadek Ahmed, Rana M. ElBishbishy, Mohamed A. Sadek, Osama Saher

**Affiliations:** 1 Department of Pharmaceutics and Industrial Pharmacy, Faculty of Pharmacy, Cairo University, Cairo, Egypt; 2 Department of Pharmacology and Toxicology, Faculty of Pharmacy, Cairo University, Cairo, Egypt; 3 Department of Laboratory Medicine, Karolinska Institute, Stockholm, Sweden; 4 Department of Cellular Therapy and Allogeneic Stem Cell Transplantation (CAST), Karolinska University Hospital Huddinge and Karolinska Comprehensive Cancer Center, Stockholm, Sweden

**Keywords:** cisplatin-induced nephrotoxicity, curcumin, mesangial uptake, quatsomes, renal biomarkers

## Abstract

The kidney plays a critical role in metabolite excretion, fluid regulation, and homeostasis, yet remains highly vulnerable to structural and functional disorders. Kidney diseases represent a major global health burden, often progressing to chronic complications due to the limited efficacy and poor selectivity of conventional therapies, which are frequently associated with systemic toxicity. Accordingly, the development of targeted drug delivery systems is essential to improve therapeutic outcomes. In this study, advanced quatsomes were developed as a kidney-targeted nanosystem to enhance the delivery of curcumin, a natural polyphenolic compound with potent antioxidant and nephroprotective properties. The system was composed of di-dodecyl-dimethyl-ammonium bromide (DDAB), cholesterol, limonene, hyaluronic acid (HA) and surfactants, and was fabricated using the ethanol injection method. A 2^3^ factorial design was employed to optimize formulation variables, including DDAB:cholesterol ratio, limonene:drug ratio, and surfactant concentration. The optimized formulation (desirability = 0.950) exhibited high entrapment efficiency (87.80%), nanosized vesicles (120.55 nm), and a positive surface charge (+38.30 mV). Transmission electron microscopy confirmed spherical morphology, while *in-vitro* release studies demonstrated a biphasic profile. The formulation also showed good physicochemical stability and enhanced antioxidant activity. Mechanistically, passive targeting via nanoscale size and cationic charge facilitated interaction with the glomerular filtration barrier and mesangial uptake, while limonene improved vesicle deformability. HA functionalization further enabled CD44-mediated active targeting. *In vivo*, the optimized system significantly reduced serum creatinine and blood urea nitrogen levels in a cisplatin-induced nephrotoxicity model, with histological evidence of renal protection. Overall, the developed quatsomes demonstrate promising potential as an efficient renal-targeted nanocarrier.

## Highlights

- Advanced Quatsomes nanocarriers were successfully developed and optimized for renal-targeted therapy.
- Optimized formula showed high entrapment efficiency (87.8%), nano-size (120.55 nm), and stable positive charge (+38.3 mV).
- TEM imaging confirmed spherical vesicles, while *in-vitro* release followed biphasic Higuchi kinetics and stability was maintained for 3 months.
- Quatsomes demonstrated a remarkable (∼3.8-fold) enhancement in antioxidant activity, reducing the IC_50_ from 10.99 μg/mL to 2.90 μg/mL
- Synergistic mechanistic advantages are achieved through the nanoscale size, cationic DDAB, and limonene, which collectively may enhance mesangial uptake, promote glomerular interaction, and facilitate deeper renal cellular penetration. In addition, hyaluronic acid functionalization, due to its known affinity for CD44 receptors, may further contribute to cellular uptake and renal localization.
- *In vivo* evaluation demonstrated significant protection against cisplatin-induced nephrotoxicity, with reduced SCr and BUN and preserved renal histology.

## Introduction

The kidney is a highly specialized organ essential for systemic homeostasis. In addition to its primary role in excreting metabolic wastes, it contributes to fluid and electrolyte balance, acid–base regulation, and vitamin D activation [1]. It also has endocrine activity through the secretion of renin, a regulator of blood pressure, and erythropoietin, which is critical for red blood cell synthesis [2]. Structurally, the kidney is composed of the cortex, medulla, and renal pelvis [3]. Its functional unit, the nephron, consists of the glomerulus and renal tubules embedded in a dense vascular network, coordinating filtration, reabsorption, and secretion [4, 5]. Due to its central role in filtration and waste elimination, the kidney is highly vulnerable to toxic insults. Nephrotoxicity is frequently observed with a range of therapeutic agents, particularly anticancer drugs [6]. Depending on the severity, renal injury may be presented as acute kidney injury (AKI) or, if left untreated, might advance to chronic kidney disease (CKD), which can ultimately result in renal failure, dialysis, or transplantation [7, 8]. In either case, common pathological manifestations are evident in the form of glomerulonephritis, fibrosis of the renal tubules, and/or diabetic nephropathy [9, 10]. These conditions impose a growing global health and economic burden.

Among nephrotoxic agents, cisplatin remains a critical example. Despite its potent anticancer efficacy, its clinical use is limited by renal toxicity, characterized by elevated serum creatinine and urea levels [11, 12]. Cisplatin enters the kidney’s proximal tubular cells through two transporters; copper transporter 1 and organic cation transporter 2 [13]. It is then activated to bind to the DNA molecules, thus locking DNA unwinding and hindering its replication. This mechanism is necessary for its anticancer effect, yet it causes cytotoxicity in the highly up taking kidney cells [14]. In addition, cisplatin generates reactive oxygen species which exerts a direct cytotoxic action on the kidney cells and promotes inflammation as well as apoptosis [15]. The prevalence of cisplatin-induced acute nephrotoxicity is around 20–30% of the patients [16]. Current therapies for kidney diseases and drug-induced nephrotoxicity provide only partial benefits and are often associated with additional side effects [17]. Conventional drugs are often constrained by poor renal selectivity, systemic distribution, and dose-limiting toxicities, which reduce treatment adherence and compromise long-term outcomes. These drawbacks highlight the urgent need for advanced approaches that can selectively target renal tissues, maximize therapeutic benefits at lower doses, and minimize systemic side effects [18, 19].

Within this framework, a newly engineered Quatsomes-based nanocarrier was developed for targeted kidney delivery. This system utilizes di-dodecyl-dimethyl-ammonium bromide (DDAB), a cationic quaternary ammonium surfactant, which serves as the fundamental structural component responsible for vesicle formation [20]. Furthermore, cholesterol, which intercalates within the bilayer to confer rigidity and stability. Cholesterol acts synergistically to prevent premature leakage during circulation, ensuring that the nanosystem remains intact until reaching its renal target [21]. Moreover, limonene, a natural monoterpene incorporated as an edge activator to improve membrane flexibility and drug entrapment and an additional surfactant that stabilizes the colloidal dispersion and enhance drug solubilization [20, 22]. Synergistically, these components self-assemble into nano-sized, spherical quatsomes with uniform morphology, high entrapment efficiency, and a strongly positive surface charge. Beyond their structural integrity, the physicochemical properties of these quatsomes are purposefully tailored to enhance kidney targeting through multiple mechanisms. The cationic nature of DDAB confers a positive zeta potential, enabling strong electrostatic interactions with the negatively charged glomerular basement membrane and tubular epithelium, thereby favoring renal accumulation [8]. The optimized nano-scale size facilitates preferential uptake by mesangial cells, which are specialized in nanoparticle internalization within the glomerulus [23]. Furthermore, the incorporation of limonene not only enhances bilayer elasticity but also improves cellular permeability and supports a sustained drug release profile once internalized into renal tissue. In parallel, hyaluronic acid (HA) functionalization introduces an active targeting dimension through its high affinity for CD44 receptors, which are abundantly expressed on mesangial cells. This ligand–receptor interaction is anticipated to promote selective cellular recognition and receptor-mediated endocytosis, thereby enhancing the localization and retention of the vesicular system within mesangial compartments and ultimately improving renal targeting efficiency. Collectively, this advanced platform combines structural stability with tailored physicochemical features to achieve efficient, selective, and safe kidney-targeted drug delivery.

Curcumin (Cur), a naturally occurring polyphenolic compound isolated from Curcuma longa, has garnered extensive research attention owing to its diverse therapeutic potential. In addition to its strong antioxidant activity, curcumin exhibits notable anti-inflammatory, antifibrotic, and hepatoprotective effects [24]. Cur also exerts a notable nephroprotective effect, primarily by modulating oxidative stress pathways and regulating biomolecules involved in purine metabolism and protein catabolism. These mechanisms collectively help preserve renal structure and function under pathological conditions [25–27]. However, the therapeutic potential of curcumin is significantly hindered by its physicochemical limitations, including extremely poor water solubility, unstable in the alkaline environments, accelerated metabolic clearance, and consequently, very low bioavailability in systemic circulation [28]. To overcome these barriers, increasing attention has been directed toward nanocarrier-based delivery systems, which provide a means to protect curcumin from premature degradation, improve solubility, enhance absorption, and prolong circulation time. These findings underscore the importance of rational nanocarrier design in quatsomes to unlock the full nephroprotective potential of curcumin.

The current work innovate a new renal-targeted vesicular module that combines the structural stability, nanoscale size for mesangial uptake, and controlled release properties to enhance drug delivery to the kidney. By overcoming the inherent limitations of free curcumin, this approach emphasizes the potential of quatsomes as a promising and translational approach for preventing and managing drug-induced nephrotoxicity.

## Materials and methods

### Materials

Curcumin (Cur, 95% purity) has been sourced through Fisher Scientific International, Inc. (Massachusetts, USA). Cholesterol, Span 80, cisplatin, HA, limonene, and the cationic surfactant di-dodecyl-dimethyl-ammonium bromide (DDAB) were procured through Sigma-Aldrich (St. Louis, MO, USA). Dialysis membranes with a molecular weight cut-off of 14,000 Da were also purchased through Sigma-Aldrich. In addition, disodium hydrogen phosphate, potassium dihydrogen phosphate, sodium chloride, and absolute ethanol (95%) were supplied by El Nasr Pharmaceutical Chemicals Company (Cairo, Egypt). The remaining solvents and reagents that used in the study were analytical grade and used as received.

### Experimental animals and ethical handling

Healthy adult male Wistar rats (200–250 g, 10–12 weeks old) were acquired from the National Organization for Drug Control and Research (NODCAR, Giza, Egypt) and allowed to acclimatize for 1 week under standardized housing conditions at the Faculty of Pharmacy, Cairo University (FOPCU). Housing was properly maintained at a temperature of 25 ± 2 °C and relative humidity of 60 ± 10%, and the animals were subjected to a 12 h light/12 h dark cycle (6 a.m.–6 p.m.). Additionally, animals had free water supply and a standard pellet chow diet throughout the study. All experimental protocols involving animals were conducted in strict accordance with internationally accepted ethical standards, including the NIH Guide for the Care and Use of Laboratory Animals and the ARRIVE reporting guidelines. Ethical approval for the study was granted by the Research Ethics Committee of the Faculty of Pharmacy, Cairo University (Approval No. PT 4013), ensuring adherence to principles of animal welfare and responsible research conduct.

### Design of experiments and factorial approach

In the present work, the development of curcumin-loaded advanced Quatsomes was designed and optimized in a 2^3^ factorial model with the aid of Design-Expert® software (Stat-Ease, Inc., Minneapolis, MN, USA). It was selected due to its efficiency in evaluating multiple formulation factors simultaneously while minimizing the number of required experiments [29]. Based on preliminary investigations, three critical variables were identified as independent factors: the DDAB-to-cholesterol molar ratio, limonene-to-drug ratio, and surfactant concentration % w/v (Factors A, B, and C; respectively). All factors were studied at two Different levels, enabling comprehensive statistical modeling and interaction analysis. To identify the optimal formulation, the selected parameters of entrapment efficiency (EE%), particle size (PS), poly-dispersity index (PDI), and zeta potential (ZP) (Y_1_, Y_2_, Y_3_, and Y_4_; respectively) were optimized to obtain vesicles with high EE%, nanoscale dimensions, uniform distribution, and optimum colloidal stability. Factorial matrix, with levels of factor and applied desirability Limitations for each response, is summarized in Table 1. All designed formulations were successfully prepared and characterized, with their experimental outcomes compiled in Table 2 [29, 30].

**TABLE 1:** Factorial scheme overview: Parameters, metrics, and research objectives.

Factor (independent variable)	Level	
	−1	+1
A: DDAB: Cholesterol ratio B: Limonene: Drug ratio C: surfactant concentration (%w/v)	1.5:1 3:1 0.4	3:1 6:1 0.8

Response (dependent variable)	Desirability constraints	
Y1: EE % Y2: PS (nm) Y3: PDI Y4: ZP (absolute value) (mV)	Maximize Minimize Minimize Maximize	

Abbreviations: DDAB, didodecyldimethylammonium bromide; EE %, percent entrapment efficiency; PDI, poly-dispersity index; PS, particle size; ZP, zeta potential.

**TABLE 2:** Measured Attributes of Curcumin-Loaded Quatsomes Formulations (n = 3, mean ± SD).

Formula	Factors						
	A: DDAB: cholesterol ratio (w/w)	B: Limonene: Drug ratio (w/w)	C: surfactant concentration (%w/v)	Y1: EE % (mean ± SD)	Y2: PS (nm) (Mean ± SD)	Y3: PDI (mean ± SD)	Y4: ZP (mV) (Mean ± SD)
F1	1.5:1	3:1	0.8	72.40 ± 3.43	130.85 ± 0.92	0.33 ± 0.08	24.40 ± 2.12
F2	3:1	6:1	0.4	83.17 ± 1.70	132.95 ± 7.01	0.31 ± 0.06	32.80 ± 2.83
F3	1.5:1	6:1	0.4	80.76 ± 3.35	132.40 ± 4.67	0.27 ± 0.02	24.15 ± 0.92
F4	1.5:1	3:1	0.4	66.07 ± 2.52	143.00 ± 2.55	0.17 ± 0.05	22.35 ± 2.47
F5	3:1	6:1	0.8	87.80 ± 3.72	120.55 ± 2.47	0.26 ± 0.08	38.30 ± 0.28
F6	3:1	3:1	0.4	69.09 ± 4.92	137.80 ± 4.53	0.35 ± 0.08	25.00 ± 1.41
F7	3:1	3:1	0.8	77.02 ± 2.65	128.90 ± 0.85	0.35 ± 0.14	33.60 ± 1.41
F8	1.5:1	6:1	0.8	85.93 ± 2.90	127.90 ± 1.70	0.32 ± 0.01	27.05 ± 0.35

Abbreviations: DDAB, didodecyldimethylammonium bromide; EE %, percent entrapment efficiency; PDI, poly-dispersity index; PS, particle size; ZP, zeta potential.

Curcumin used was 10 mg in all formulations.

### Formulation of advanced quatsomes

Curcumin-loaded advanced Quatsomes were fabricated using a modified ethanol injection technique, a well-established method for producing nano-vesicular systems with controlled size and high reproducibility [30, 31]. In brief, both curcumin (10 mg), cholesterol (30 mg), as well as predetermined amounts of DDAB, limonene, and span 80, were all dissolved in 5 mL absolute ethanol (organic phase). The mixture was placed in a thermostatically controlled water bath (Crest Ultrasonics Corp., Trenton, USA) at a temperature of 60 °C, until complete dissolution. Simultaneously, the aqueous phase consisting of Milli-Q water (preheated to 60 °C) was prepared. Milli-Q water was specifically chosen over double-distilled water to avoid ionic contaminants and trace impurities, which could potentially interfere with vesicle self-assembly, surface charge uniformity, and stability [32]. Under continuous magnetic stirring (600 rpm) using an MSH-20D magnetic stirrer (GmbH, Berlin, Germany), the ethanolic phase was slowly injected into the aqueous phase at a ratio of 1:2 (v/v), triggering spontaneous self-assembly of quatsomal nanovesicles. The injection was performed at a controlled rate of ∼0.5 mL/min using a 22G needle (0.41 mm inner diameter). Stirring was then maintained at 1000 rpm for 2 h to ensure complete ethanol removal, yielding a clear, homogeneous, and stable dispersion, with completion confirmed by the absence of ethanol odor. To further refine vesicle size and improve homogeneity, the dispersions were sonicated for 5 min (Crest Ultrasonics Corp., Trenton, USA). Finally, formulations were allowed to equilibrate at room temperature and stored at 4 °C until further physicochemical and biological evaluations [33, 34].

### *In Vitro* assessment of curcumin-loaded quatsomes

#### Quantification of curcumin entrapment

The encapsulation efficiency (EE%) of curcumin within the quatsomal system was determined indirectly by quantifying the un-encapsulated drug in the aqueous phase. Briefly, 1 mL of freshly prepared formulation was ultracentrifuged at 21,000 rpm (Sigma 3K30, Sigma Laborzentrifugen GmbH, Germany) for 60 min at 4 °C to separate the free drug from the vesicular fraction [35]. The supernatant containing the non-entrapped curcumin was collected and appropriately diluted for spectrophotometric analysis at λmax 425 nm using a UV–visible spectrophotometer (Shimadzu UV-1601 PC, Kyoto, Japan) [35]. Quantification was performed using a validated calibration curve (R^2^ = 0.9995, n = 3). A blank containing all formulation components without curcumin was used to correct for any potential interference, and its absorbance was subtracted from sample readings. Encapsulation efficiency was then calculated using Equation 1 [36]:

$$EE\;\%=\frac{\left(Total\;amount\;of\;Cur-Unentrapped\;Cur\right)}{Total\;amount\;of\;Cur}\;\times \;100$$ (1)

#### Assessment of particle size (PS) and surface potential (SP)

Particle size, polydispersity index (PDI), and zeta potential of curcumin-loaded quatsomes were evaluated to assess their colloidal properties. Measurements were performed using dynamic light scattering (DLS) with a Nano Zetasizer (Model ZEN3600, Malvern Instruments Ltd., UK) at 25 °C and a fixed scattering angle of 173°. Freshly prepared samples (50 µL) were diluted 1:100 with Milli-Q water to obtain a translucent, non-aggregated suspension suitable for analysis [37]. PDI was used to assess size distribution homogeneity, where lower values indicate a more uniform vesicular population [38]. Zeta potential was determined from electrophoretic mobility using the same instrument to evaluate surface charge and predict colloidal stability [39]. All measurements were performed in triplicate, and results were expressed as mean ± SD to ensure reproducibility [40, 41].

### Optimization of quatsomes

The optimization of the developed quatsomes was performed using Design-Expert® software (Stat-Ease Inc., Minneapolis, USA) to obtain a formulation with optimal physicochemical properties. The objective was to achieve vesicles with minimal particle size (PS), low polydispersity index (PDI), high encapsulation efficiency (EE%), and maximum absolute zeta potential (ZP), thereby ensuring improved stability and reduced aggregation tendency. The effects of formulation variables on the selected responses were analyzed using analysis of variance (ANOVA) [22]. A numerical optimization approach based on desirability function was applied, where each response was transformed into a scale ranging from 0 (undesirable) to 1 (highly desirable). These were then combined into a composite desirability index to allow simultaneous optimization of all parameters. The formulation with the highest overall desirability was selected as the optimized system and subjected to confirmatory experiments. The predicted and experimental values were compared to evaluate model reliability, and the percentage deviation was calculated [42, 43]. Minimal deviation confirmed the robustness and reproducibility of the optimization process (Equation 2), supporting the rational design of a stable nanocarrier for effective renal drug delivery [44, 45].

$$\%\text{ Deviation}=\left(\left|\text{Predicted value}-\text{Observed value}\right|/\left(\text{Observed value}\right)\right)\times 100$$ (2)

### Characterization of the optimized formula

HA was subsequently added to the optimized quatsome formulation to enhance renal targeting through active CD44-mediated recognition on mesangial cells. HA was incorporated by slowly sprinkling it into the quatsome dispersion under continuous stirring at room temperature until a uniform and homogeneous mixture was obtained, ensuring stable surface functionalization without compromising vesicle integrity.

#### TEM for morphological assessment

The ultrastructural features of the optimized curcumin-loaded Quatsomes were investigated using transmission electron microscopy (TEM, JEOL, Tokyo, Japan). To ensure proper dispersion, the optimized formula was initially diluted, and a small droplet was precisely spread over a carbon-coated copper grid [46, 47]. The droplet was left to air-dry under ambient conditions, after which negative staining with phosphotungstic acid solution was employed to enhance image contrast and clearly define vesicular boundaries. Once fully dried, the prepared grids were scanned at an accelerating voltage of 80 kV [48, 49].

#### FTIR spectral analysis

Fourier Transform Infrared Spectroscopy (FTIR) was performed to investigate possible molecular interactions between curcumin and the formulation components, as well as to confirm its successful incorporation within the optimized quatsomal system. Spectra were recorded for pure curcumin, cholesterol, DDAB, and the optimized formulation. Briefly, accurately weighed samples were finely ground with dried potassium bromide (KBr) and compressed into pellets prior to analysis using a Bruker FTIR spectrometer (Model 22, Coventry, UK) [50, 51]. Measurements were conducted at room temperature over a spectral range of 4,000–500 cm^−1^. The characteristic absorption bands of each individual component were compared with those of the optimized formulation to identify any shifts in peak position or changes in intensity [22].

#### *In vitro* release and the kinetic interpretation

The *in vitro* release profile of the optimized curcumin-loaded quatsomal formulation was evaluated and compared with a plain drug suspension using the dialysis bag method. The curcumin suspension was prepared at an equivalent drug concentration to the optimized formulation and dispersed in water containing 0.5% w/v carboxymethyl cellulose (CMC) as a suspending agent. Dialysis membranes (molecular weight cut-off 12,000–14,000 Da) were pre-soaked overnight in phosphate-buffered saline (PBS, pH 7.4). Accurately weighed samples of the optimized formulation or free curcumin suspension (equivalent to 1 mg drug) were placed into the dialysis bags and sealed [52]. The bags were then immersed in 50 mL of PBS (pH 7.4) maintained at 37 ± 0.5 °C in a thermostatically controlled shaking water bath at 100 rpm to simulate physiological conditions. At predetermined time intervals (0.5, 1, 2, 4, 6, and 8 h), aliquots were withdrawn from the release medium and immediately replaced with fresh PBS to maintain sink conditions [53]. The released drug was quantified spectrophotometrically at λmax 425 nm, and cumulative percentage release was plotted against time. To elucidate the release mechanism, the data were fitted to kinetic models including zero-order, first-order, Higuchi, and Korsmeyer–Peppas equations. The best-fit model was selected based on the highest correlation coefficient (R^2^), providing insight into the dominant drug release mechanism from the quatsomal system [21, 54].

#### Antioxidant assay

Free radical scavenging activity was used to assess the antioxidant capacity of the enhanced quatsomal formulation using the 2,2-diphenyl-1-picrylhydrazyl (DPPH) assay. The assay was performed over a concentration range of 5–1000 μg/mL for both the tested samples and the reference standard (ascorbic acid), with all measurements conducted in triplicate and results expressed as mean values. A freshly prepared methanolic DPPH solution (0.004% w/v), stored at 10 °C in the dark, was used as the free radical source. In each experiment, 3 mL of DPPH solution was mixed with 40 µL of sample solution prepared in methanol. Absorbance was immediately recorded using a UV–visible spectrophotometer (Milton Roy, Spectronic 1201) at 515 nm, and continuous readings were taken at 1-min intervals until a stable absorbance was achieved (approximately 16 min) [55]. The antioxidant capacity was quantified based on percentage inhibition (PI) of DPPH radicals and IC_50_ values, where lower IC_50_ values indicate higher antioxidant potency. Statistical analysis was performed using ANOVA followed by L.S.D. test to assess significant differences between groups. The DPPH radical’s percentage inhibition (PI) was determined according to Equation 3 [56, 57]:

$$PI=\frac{\left(AC-AT\right)}{AC}\;\times \;100$$ (3)

AC is the control initial absorbance and AT is the test sample absorbance after 16 min [55].

#### Stability evaluation

To verify the short-term stability of the optimized quatsomal formulation, all samples were preserved in a well-sealed, amber-coloured glass vials in a refrigerated atmosphere (5 ± 3 °C) for a duration of 3 months. The study was designed to ensure that the nanosystem maintained its structural integrity and therapeutic performance throughout storage. At the end of the storage period, the formulation was reassessed for its critical physicochemical characteristics, including PS, PDI, ZP, and EE%. These parameters were statistically compared to those of the freshly prepared sample using one-way ANOVA. In addition to numerical analyses, visual inspection was conducted to check for physical instability indicators such as precipitation, aggregation or colour change [58, 59]. The *in vitro* drug release behaviour of the formulation after storage was reassessed using the same experimental conditions applied in the initial study. To quantitatively compare the release patterns obtained before and after storage, the similarity factor (f_2_) was determined using Equation 4 [35]:

$$f_{2}=50.\log{\left[1+\left(\frac{1}{n}\right)\sum_{t=1}^{n}{\left(R_{t}-T_{t}\right)}^{2}\right]}^{-0.5}100$$ (4)

the R_t_ and T_t_ represent cumulative percentage of curcumin released at each sampling time prior to and following storage, respectively, and n is the sampling points number. The calculated ƒ_2_ value between 50 and 100 was considered evidence of comparable release behavior, thereby confirming the stability of the formulation during refrigerated storage [29].

### *In vivo* analysis

#### General experimental procedures

Twenty-four rats were randomly distributed on four groups (n = 6/group). Group I rats were orally given 0.2 mL saline with 100 µL of Tween 80 daily (negative control). Additionally, groups II, III, and IV, each received a single i.p. dose of cisplatin (5 mg/kg) [60]. Group II was left untreated (the model group), while group III was treated for 10 days with 25 mg/kg standard curcumin p.o. dissolved in normal saline with 100 µL of Tween 80 (curcumin-treated group) [61]. In line, group IV was administered an oral nano formulation of curcumin (25 mg/kg) (nano formulation-treated group).

#### Sample collection

To collect the blood samples upon completion of the treatment regimen, ketamine and xylazine were used to anaesthetize the rats with a dose of 50 mg/kg i.p. and 10 mg/kg i.p., respectively [62]. In a Wasserman tube, blood was sampled from the eye canthus and stored in an upright position till centrifugation. Centrifugation was carried out at a 4 °C, 3000 rpm rotation in each 15 min cycle. After centrifugation, a non-hemolyzed, clear supernatant serum was obtained and preserved at −20 °C until biochemical testing. Subsequently, animals were sacrificed through cervical dislocation, their kidneys were immediately harvested and fixed in 10% formalin (v/v) for histological inspection.

#### Biochemical parameters

Serum creatinine (SCr) level was examined using BioAssay Systems' creatinine ELISA assay kit (cat#: DICT-500). In parallel, urea level was assessed colorimetrically by BioAssay Systems' urea ELISA assay kit (cat#: DIUR-500). Noteworthy, test procedures were consistent with the steps stated in the manufacturer’s guide. Both kits were purchased from BioAssay Systems (CA, USA).

#### Histological examination

Gross morphology of the kidney was examined by dyeing with H&E stain. Tissues fixed in formalin were carefully dehydrated using serial dilutions of alcohol. The dehydrated tissues were then embedded in paraffin and sliced into 3-µm sections. After that, xylene was employed to remove paraffin from the embedded slices, which were washed with hematoxylin eosin gradient ethanol for a period of 5 min. Once dehydrated with conventional ethanol, slices were sealed, investigated, and lesions were assessed and scored semi-quantitatively to describe the extent of pathological alteration [57].

#### Statistical analysis

Statistical evaluations were carried out using GraphPad Prism software (version 9; San Diego, CA, USA) [63]. Data distribution was assessed for normality using the Shapiro–Wilk test, and variance equality was examined with the Brown–Forsythe test. When parametric assumptions were met, differences among groups were analyzed using one-way analysis of variance (ANOVA), followed by Tukey’s multiple comparison test. A p-value of less than 0.05 was considered statistically significant for all analyses [64, 65].

## Results

### Analysis of factorial design

To systematically optimize the curcumin-loaded quatsomes for kidney-targeted delivery, a 2^3^ full factorial design was employed. This statistical approach enabled simultaneous evaluation of three critical formulation factors: the DDAB: cholesterol ratio (Factor A), limonene: drug ratio (Factor B), and surfactant concentration (Factor C). Preliminary screening was performed to define the high and low levels of each variable, ensuring both feasibility and relevance to renal-targeted delivery goals [42]. For all responses, the variation between predicted and adjusted R^2^ values were below 0.2, while adequate precision values exceeded the recommended threshold of 4, confirming excellent predictive reliability across the design space [66] (Table 3).

**TABLE 3:** Statistical examination of experimental outcomes.

Response	R^2^	Adjusted R^2^	Predicated R^2^	Adequate precision	Significant factors
EE %	0.9897	0.9820	0.9588	29.34	A,B,C
PS (nm)	0.9350	0.8863	0.7402	12.31	B,C
ZP (mV)	0.9074	0.8380	0.7297	10.42	A,C

Abbreviations: EE %, percent entrapment efficiency; PDI, poly dispersity index; PS, particle size; ZP, zeta potential.

#### Statistical assessment of EE%

**FIGURE 1:**
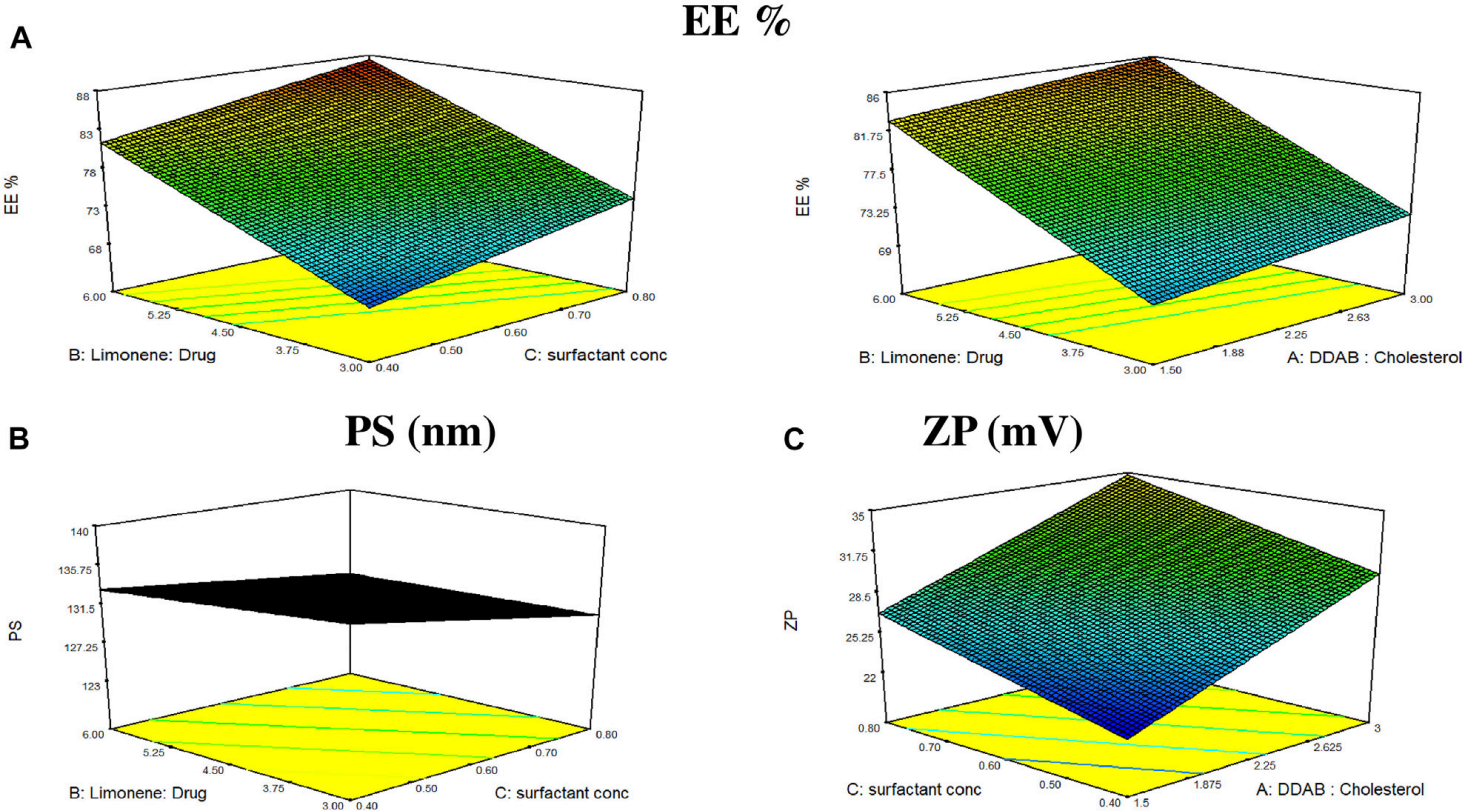
Response 3D plots for the effect of DDAB: Cholesterol ratio (Factor A), Limonene: Drug ratio (Factor B) and surfactant concentration (%w/v) (Factor C) on **(A)** entrapment efficiency percentage (EE%), **(B)** particle size (PS) and **(C)** zeta potential (ZP), highlighting the significant formulation variables influencing each response.

Efficient drug encapsulation represent a cornerstone of nanosystem design, where high retention at the site of application is required to maximize therapeutic outcomes [67]. In the present work, the entrapment efficiency (EE%) of the formulated quatsomes ranged between 66.07 ± 2.52% and 87.80 ± 3.72% (Table 2), confirming their strong capacity to accommodate the lipophilic drug within the vesicular matrix. Statistical analysis demonstrated that all investigated formula variables showed a significant positive effect (*p* < 0.05) on EE% (Figure 1A), emphasizing the importance of each factor in modulating drug entrapment. The relationship is represented by the following coded equation:

EE%=77.78+1.49A+6.64B+3.01C

#### Statistical assessment of PS

Particle size is a crucial parameter in nanocarrier design, as it dictates biological fate, bio-distribution, and ultimately therapeutic efficacy. In particular, maintaining vesicles within the nanometer range is vital for mesangial uptake, where particles below ∼150 nm are more efficiently internalized, ensuring targeted renal accumulation and enhanced pharmacological action [8]. The particle size (PS) of the formulated quatsomes ranged between 120.55 ± 2.47 and 143.00 ± 2.55 nm (Table 2), depositing them within the optimal nanoscale domain for mesangial delivery. The statistical analysis confirmed that the model was significant, with factors B (limonene: drug ratio) and C (surfactant concentration) exerting negative and significant influences on PS (Figure 1B), whereas factor A (DDAB: cholesterol ratio) showed no meaningful effect. The observed relationship is described by the following coded equation:

PS=131.79 – 1.74A – 3.34B – 4.74C

#### Statistical assessment of PDI

Poly-dispersity index (PDI) is widely recognized as a critical descriptor of nanoparticle size distribution, reflecting the homogeneity and reproducibility of vesicular systems. Values approaching zero denote highly uniform dispersions, while those closer to one imply heterogeneous populations. In pharmaceutical nanocarrier development, PDI values below 0.5 are generally considered acceptable, indicating a narrow size distribution that supports consistent drug release and predictable biological performance [49]. In the current work, PDI values of the formulated quatsomal formulations ranged from 0.17 ± 0.05 to 0.35 ± 0.14 (Table 2). This consistently low range highlights the ability of the preparation method to generate vesicles with uniform particle size distribution, a desirable feature for ensuring stability, reproducibility, and efficient cellular uptake. Statistical analysis using ANOVA demonstrated that PDI model was not statistically significant (*p* > 0.05), and none of the investigated formulation variables exerted a meaningful influence on PDI values. Although the parameter was excluded from the optimization criteria due to its lack of statistical sensitivity, the consistently favorable PDI values obtained reinforce the robustness of the fabrication process and confirm the reliability of the nanosystem for drug delivery applications.

#### Statistical assessment of ZP

Zeta potential (ZP) is a pivotal parameter in evaluating the electrostatic stability of nano-vesicular systems, as it reflects the magnitude of repulsive forces between particles. Sufficiently high absolute ZP values enhance colloidal stability by preventing aggregation and flocculation, ensuring a uniform dispersion suitable for pharmaceutical applications. Typically, nanosystems with surface charges exceeding ±20 mV are considered electrostatically stable due to effective inter-vesicular repulsion [68]. In this study, the prepared quatsomal formulations exhibited ZP values ranging from 22.35 ± 2.47 to 38.30 ± 0.28 mV (Table 2), confirming their stability. Regression analysis of the experimental data yielded the following predictive equation:

ZP=28.46+3.97A+2.12B+2.38C

where Factor A represents the DDAB: cholesterol ratio, Factor B corresponds to the limonene: drug ratio, and Factor C denotes the surfactant concentration. Among these, both Factor A (DDAB: cholesterol ratio) and Factor C (surfactant concentration) exerted statistically significant positive effects (*p* < 0.05) on the magnitude of ZP, suggesting that higher proportions of cationic lipid and surfactant strengthened the surface charge and thus improved dispersion stability. Figure 1C illustrates 3D-plots of the significant factors.

### Optimization of quatsomes

The optimization of curcumin-loaded quatsomes was carried out using Design-Expert® software, which applies a desirability function to integrate multiple formulation responses into a single score ranging between 0 (least favorable) and 1 (most favorable) [69]. Based on the software-guided analysis, the optimal formulation was identified at a DDAB: cholesterol at a 3:1 ratio (Factor A), a limonene: drug at a 6:1 ratio (Factor B), and a surfactant concentration of 0.8% (Factor C). This composition achieved an excellent overall desirability score of 0.950, with EE% of 87.80 ± 3.72%, PS of 120.55 ± 2.47 nm, and ZP of +38.30 ± 0.28 mV. The close agreement between predicted and experimental outcomes, with deviations not exceeding 5%, validated the robustness and predictive accuracy of the model. Collectively, these findings confirm that the optimized quatsomes possess balanced physicochemical attributes, strong encapsulation capability, and high colloidal stability, making them a promising nanocarrier system for kidney-targeted curcumin delivery [70].

### Detailed examination of the enhanced formula

The effect of hyaluronic acid (HA) incorporation on the optimized formulation was evaluated by comparing key parameters. The results showed that EE% (86.25 ± 1.10), PS (122.40 ± 4.53 nm), and ZP (+36.90 ± 0.42 mV) were not significantly changed after HA addition (p > 0.05), indicating that HA did not affect the physicochemical properties of the nanoparticles.

#### TEM for morphological assessment

**FIGURE 2:**
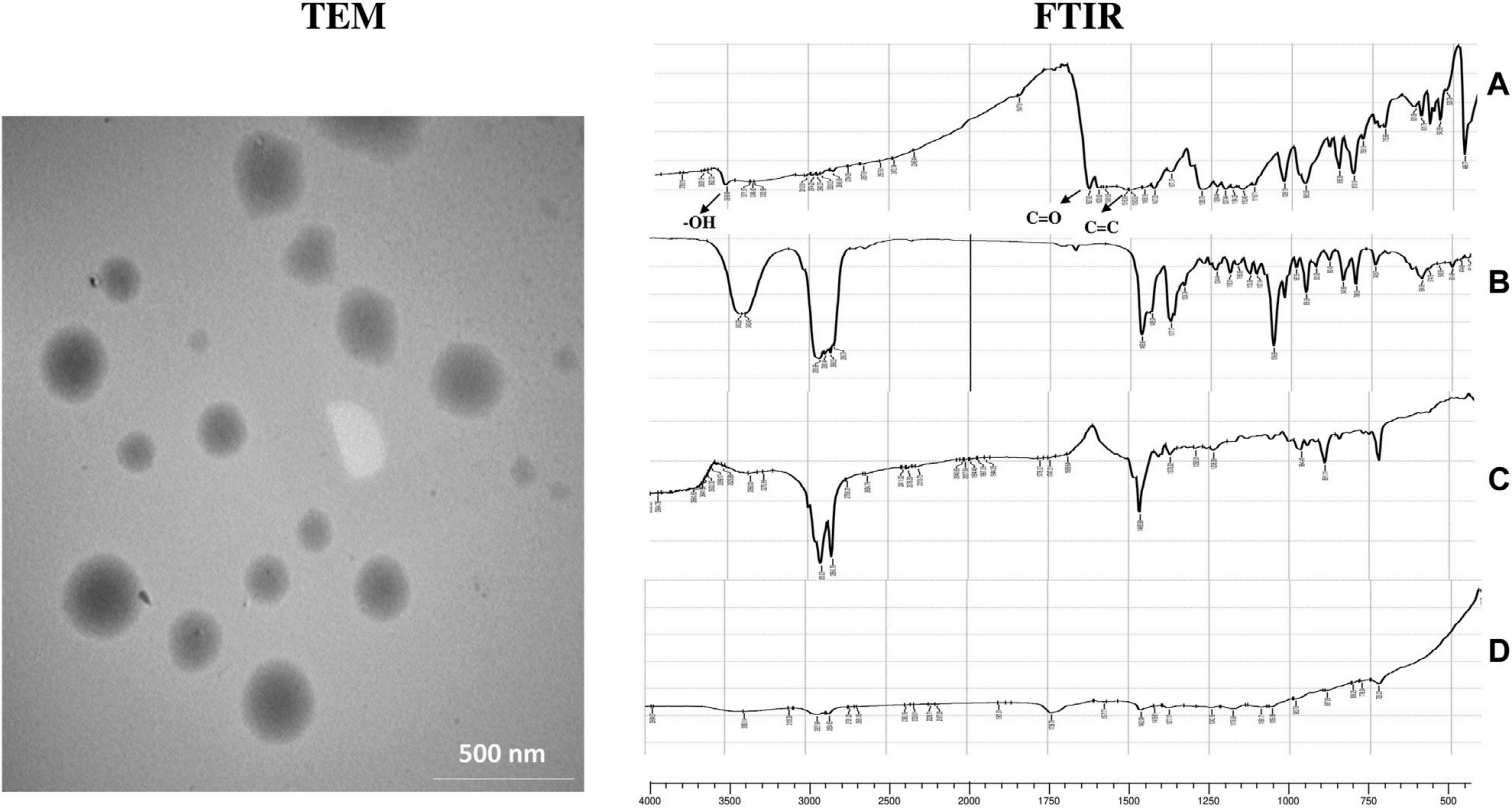
FTIR spectra of pure **(A)**Cur, **(B)**Cholesterol, **(C)** DDAB and **(D)** optimum formula. In addition to, TEM of the optimum formula, showing consistently spherical vesicle morphology and the lack of distinct curcumin peaks, indicating successful physical encapsulation within the nanocarrier.

TEM was employed to visualize the morphological features of the optimized curcumin-loaded quatsomes, providing direct evidence of their nanoscale architecture (Figure 2). The images revealed uniform, spherical vesicles with smooth surfaces and a narrow size distribution, in excellent agreement with dynamic light scattering (DLS) data. Importantly, the vesicles appeared discrete and non-aggregated, without signs of deformation, fusion, or clustering, underscoring the high colloidal stability of the system [71, 72].

#### FTIR spectral analysis

FTIR spectroscopy was conducted to evaluate the molecular compatibility of the formulation components and to confirm the successful incorporation of curcumin into the optimized quatsomal system (Figure 2). The spectrum of pure curcumin displayed characteristic vibrational bands, including a broad peak at 3510–3515 cm^−1^ corresponding to phenolic–OH stretching, a strong carbonyl (C=O) stretching signal at 1625–1630 cm^−1^, and aromatic C=C stretching vibrations at 1600 cm^−1^ and 1500–1505 cm^−1^, confirming its distinct chemical structure [58]. Cholesterol exhibited diagnostic absorption peaks including O–H stretching at 3415–3420 cm^−1^, aliphatic C–H stretching between 2,800 and 3000 cm^−1^, CH_2_ scissoring at 1460–1470 cm^−1^, CH_3_ symmetric bending at 1375–1380 cm^−1^, and C–O stretching in the fingerprint region at 1050–1150 cm^−1^, reflecting its molecular identity [73]. DDAB demonstrated characteristic methylene-related peaks at 721, 886, and 1467 cm^−1^, along with symmetric and asymmetric C–H stretching at 2,852 and 2,919 cm^−1^ [74].

#### *In vitro* release and kinetic interpretation

**FIGURE 3:**
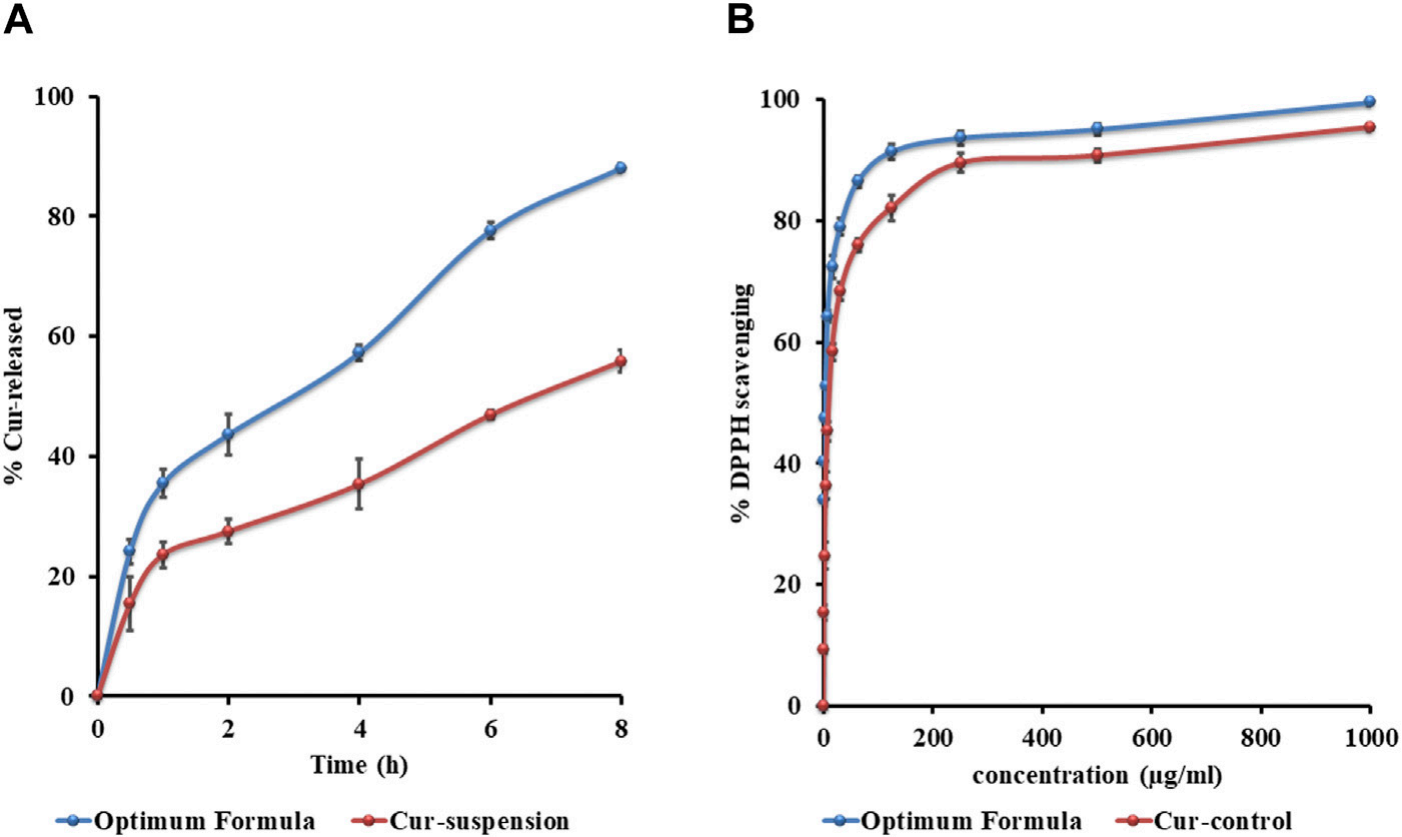
**(A)** *In vitro* release profiles **(B)** *In vitro* antioxidant activity (DPPH analysis) from optimum formula compared to that from Cur-control, demonstrating biphasic drug release and enhanced antioxidant activity, (mean ± SD, n = 3).

The release behaviour of curcumin from the optimized quatsomal formulation was systematically examined in phosphate-buffered saline (pH 7.4) to mimic physiological conditions. As illustrated in Figure 3A, the optimized nanosystem exhibited a biphasic pattern characterized by an initial rapid phase followed by a prolonged, controlled release. Approximately 44% of curcumin was liberated within the first 2 h, which can be attributed to surface-associated or loosely bound drug molecules diffusing quickly into the release medium [75]. Followed by a sustained release phase, that reflect the gradual diffusion of curcumin from the innermost hydrophobic domains of the vesicular bilayer. In contrast, the aqueous curcumin suspension displayed markedly inferior performance, underscoring the limitations imposed by curcumin’s poor aqueous solubility.

#### Antioxidant assay

The antioxidant potential of the developed quatsomes was evaluated using the DPPH radical scavenging assay, a widely recognized method for quantifying free radical neutralization. Compared with free curcumin, the nano-formulated system exhibited a striking improvement in radical scavenging efficiency (Figure 3B). Specifically, the IC_50_ value decreased from 10.99 μg/mL (free curcumin) to 2.90 μg/mL (quatsomes), corresponding to an approximate 3.8-fold enhancement in activity.

#### Stability evaluation

The stability of the optimized curcumin-loaded quatsomes was systematically assessed at 5 ± 3 °C for a period of 3 months to assess their robustness during storage. Visual inspection confirmed that the dispersion retained its original clarity without evidence of turbidity, sedimentation, or vesicular aggregation, suggesting excellent physical stability. Furthermore, no statistically significant differences (*p* > 0.05) between freshly prepared and stored samples were observed in key physicochemical attributes including entrapment efficiency (EE%), particle size (PS), and zeta potential (ZP), as presented in Table 4 [36]. These findings indicate that the nanocarrier system preserved its structural integrity throughout the storage period. Drug release studies further substantiated these results, with the release profiles of the fresh and stored formulations demonstrating high similarity (f_2_ = 67.94), confirming that the sustained release behavior of quatsomes remained unaltered post-storage [76, 77].

**TABLE 4:** Stability assessment of the optimized formula after storage.

Parameter	Fresh	Storage for 3 months at 4–8 °C	
		Value	Probability (*p*)*
EE %	87.80 ± 3.72	85.02 ± 1.57	0.432
PS	120.55 ± 2.47	117.05 ± 2.62	0.303
ZP	38.30 ± 0.28	37.20 ± 1.98	0.518

Abbreviations: EE %, percent entrapment efficiency; PS, particle size; ZP, zeta potential.

* One-way ANOVA, analysis to compare between the freshly prepared and the stored optimum formula.

### *In vivo* analysis

#### Biochemical results

**FIGURE 4:**
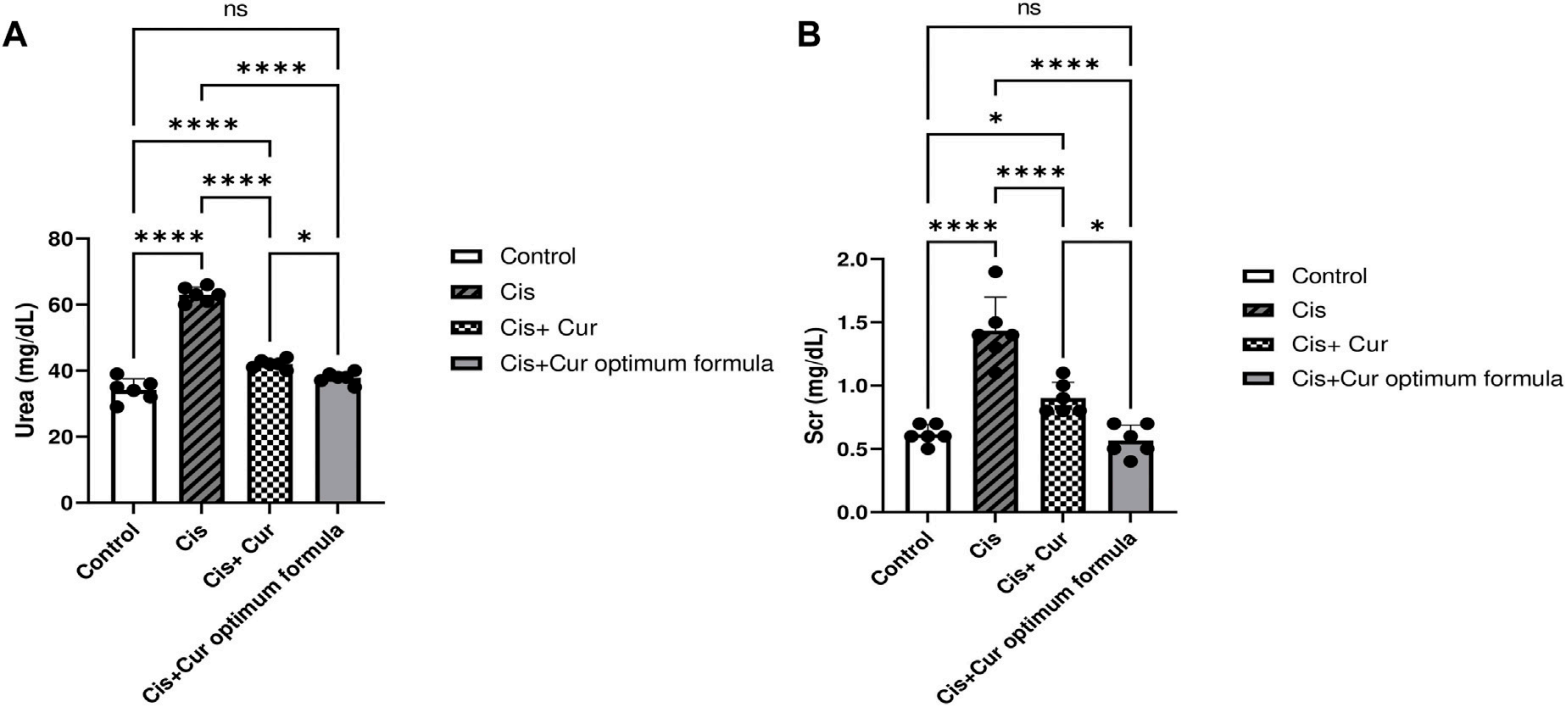
Statistical comparison of Kidney function markers **(A)** urea and **(B)** serum creatinine (SCr). Statistical significance assessed by one-way analysis of variance (ANOVA) followed by Tukey’s *post hoc* test (n = 6, asterisks indicate significant difference between compared groups, *p < 0.05, **p < 0.01, ***p < 0.001 and ****p < 0.0001). The results demonstrate that the optimized formulation significantly reduced serum creatinine and blood urea nitrogen levels, indicating enhanced nephroprotective efficacy.

Cisplatin (Cis) is a potent anticancer agent that, unfortunately, causes serious kidney injury upon administration. Its nephrotoxic effect can be assessed by measuring markers for kidney injury, such as the SCr and urea. In the current study, SCr and urea levels exhibited a huge incline upon Cis administration, reaching 2.3- and 1.8-fold, respectively, as compared to normal control rats (p < 0.0001). Administration of standard curcumin suspension slightly decreased the kidney injury indicators compared to the model group, where the SCr and urea declined by 37.2- and 33.3%, respectively (p < 0.0001). However, treatment with the optimum formula evidently reduced the SCr by 60.5% (p < 0.0001), while the urea level declined by 40% (p < 0.0001) relative to the Cis-only group, thus signifying superior nephroprotection (Figures 4A,B). Importantly, a statistically significant difference was observed between the optimum formulation and the curcumin suspension (p < 0.05), confirming the enhanced therapeutic efficacy of the optimized system.

#### Histological examination

**FIGURE 5:**
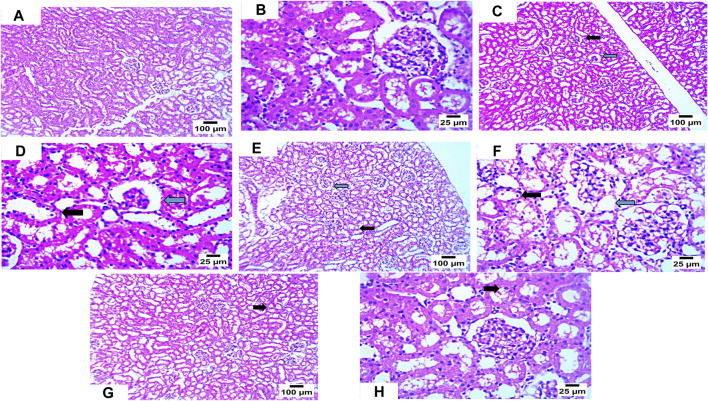
Microscopic photographs showing **(A)** normal structure of renal tubular epithelium in control group (X100), **(B)** normal structure of renal tubular epithelium in control group (X400), **(C)** severe damage in the tubular epithelium accompanied by irreversible necrobiotic changes (black arrow) and multiple collapsed glomeruli (blue arrow) in model group (X100), **(D)** severe damage in the tubular epithelium accompanied by irreversible necrobiotic (black arrow) and multiple collapsed glomeruli (blue arrow) in model group (X400), **(E)** marked expansion of the urinary space (blue arrow),along with irreversible necrobiotic alterations (black arrow) in some renal tubular epithelial cells in standard curcumin group (X100), **(F)** marked expansion of the urinary space (blue arrow),along with irreversible necrobiotic alterations (black arrow) in some renal tubular epithelial cells in standard curcumin group (X400), **(G)** minimal reversible cell damage, nuclear pyknosis(black arrow), which was observed in the epithelium of some renal tubules (X100), **(H)** minimal reversible cell damage, nuclear pyknosis (black arrow), which was observed in the epithelium of some renal tubules (X400). Lesions were assessed and scored on a scale from 0, with the least damage, to 3, which has the greatest damage. Scores were as follows: Group 1: 0; Group 2: 3; Group 3: 2; Group 4: 1.

The previously mentioned results match the histological results, where a normal structure of renal tubular epithelium was observed in the control group (Figures 5A,B). Lesions were scored based on the extent of observed damage. The score of the normal control group was zero. In contrast, the model group demonstrated severe damage in the tubular epithelium accompanied by irreversible necrobiotic changes and multiple collapsed glomeruli (Figures 5C,D). The lesion score for the model group was 3, which indicates severe damage to the kidneys induced by cisplatin. The standard curcumin group exhibited marked expansion of the urinary space, along with irreversible necrobiotic alterations in some renal tubular epithelial cells and had a lesion score of 2 (Figures 5E,F). Specimens of nano-formulated curcumin, however, received the score of 1, since they demonstrated minimal reversible cell damage and nuclear pyknosis, which was observed in the epithelium of some renal tubules (Figures 5G,H).

## Discussion

Regarding EE%, DDAB (Factor A) had a significant positive influence. Owing to its high lipophilicity (log P ≈ 11.8) [78], DDAB enhanced bilayer hydrophobicity and rigidity, minimizing drug diffusion into the aqueous medium and promoting tighter vesicle packing. This effect was further complemented by its quaternary ammonium head group, which contributed to electrostatic stabilization of the system [36]. Similarly, limonene (Factor B) improved EE% by reinforcing bilayer hydrophobicity. Its high lipophilicity (log P ≈ 4.83) and hydrocarbon backbone (C_10_H_16_) allowed better drug solubilization within the lipid domain and provided additional structural stability to the vesicles [70, 79]. The presence of limonene’s extended carbon chain also favoured hydrophobic interactions with both surfactant and drug, further supporting encapsulation. Regarding surfactant concentration (Factor C), Span 80, a low-HLB surfactant (HLB ≈ 4.3), enhanced vesicular encapsulation owing to its long unsaturated oleate chain (C_18_) [80]. This structural feature reduces the number of hydrophilic voids in the bilayer and decreases overall membrane fluidity by attenuating its amphiphilic character [81, 82]. In addition, higher surfactant concentrations improved emulsification and stabilization of the lipid matrix, expanded the volume of the hydrophobic bilayer, and facilitated the formation of a larger number of vesicles, thereby providing a more suitable housing domain for the hydrophobic drug [83]. Collectively, these findings confirm that optimizing the DDAB: cholesterol ratio, surfactant concentration, and limonene content synergistically enhances the entrapment efficiency of quatsomes. This balance of components is therefore critical for developing vesicles with superior drug encapsulation and stability, ensuring their suitability for effective renal delivery.

Regarding PS, The observed reduction in vesicle size with increasing limonene: drug ratio can be attributed to the function of limonene as an Ostwald ripening inhibitor. Limonene suppresses molecular migration within the dispersion medium, thereby reducing vesicular coalescence and preventing excessive growth. At elevated concentrations, its high lipophilicity (log P = 4.83) and long carbon backbone (C_10_H_16_) contribute to enhanced stabilization of the dispersed phase by limiting inter-vesicular interactions and maintaining a smaller hydrodynamic diameter. This stabilizing effect also arises from the increased surface-to-volume ratio achieved with higher limonene content, which minimizes aggregation and improves dispersion uniformity. The same results have been noted by Tawfik et al., who reported terpene-mediated downsizing of zolmitriptan nanovesicles, and by Albash et al., where higher terpene levels yielded smaller fenticonazole nitrate terpesomes [70, 84]. Collectively, these findings emphasize limonene’s dual role as a lipophilic membrane modifier and anti-ripening agent, making it a critical determinant in tuning vesicle size for optimized nanocarrier performance. Regarding factor C (surfactant concentration), increasing the surfactant level provided additional coverage at the vesicular interface, effectively lowering the interfacial tension between quatsomes and the aqueous medium. This interfacial stabilization prevented vesicle aggregation and promoted the formation of smaller, more uniform particles [85]. Similar findings were reported by Eldeeb et al., in cubosomal formulations of brimonidine tartrate for glaucoma and by Younes et al., in ocular delivery systems using mixed micelles, where enhanced surfactant concentration yielded smaller vesicles by controlling core swelling [86, 87].

Regarding ZP, The enhancement attributed to Factor A can be explained by the quaternary ammonium head groups of DDAB, which intensify the positive surface charge when present at higher ratios [36, 70]. Similarly, increasing surfactant levels (Factor C) likely facilitated more efficient molecular organization at the vesicle interface, reducing interfacial tension and enhancing charge expression. Furthermore, Span 80 introduced an additional stabilizing effect beyond electrostatics. Its long, unsaturated hydrocarbon chain created a steric barrier around the vesicles, physically hindering close particle–particle interactions and further reducing the likelihood of aggregation [81]. In contrast, Factor B (limonene: drug ratio) demonstrated no significant impact on ZP values (*p* > 0.05), indicating that limonene incorporation within the tested range did not substantially alter the electrostatic environment of the vesicles. Collectively, these findings confirm that the optimized quatsomal system maintained adequate surface charge across all formulations.

Regarding TEM, the structural uniformity can be attributed to the synergistic contributions of the formulation components. The cationic nature of DDAB imparted a strong positive surface charge, generating electrostatic repulsion that prevented vesicle aggregation [36]. Cholesterol played a complementary role by reinforcing bilayer rigidity and mechanical stability. Additionally, surfactant molecules contributed steric stabilization, creating a protective hydrated shell that reduced vesicle–vesicle contact [44]. It’s important to note that DLS provides the hydrodynamic diameter, representing the average size of nanoparticles in a hydrated and well-dispersed state. In contrast, TEM images particles in a dried state under vacuum and typically examines a limited number of particles, which may not fully represent the overall particle population.

Regarding FTIR, in the spectrum of curcumin-loaded quatsomes, notable attenuation or broadening of curcumin-specific peaks was observed, indicating effective physical encapsulation within the vesicular bilayer. No additional peaks or shifts suggesting chemical incompatibility were detected, confirming the molecular compatibility of the formulation components and validating the structural integrity of the optimized quatsomal system [88].

Regarding *in vitro* release, the biphasic release profile of quatsomes can be explained by their unique structural composition. The presence of surfactants, particularly span 80, enhanced hydration and solubilization of surface drug, thereby facilitating the initial burst [35]. Meanwhile, cholesterol reinforced the bilayer rigidity, restricting rapid diffusion and supporting sustained release. Cholesterol-based carriers are widely employed in drug delivery due to their biocompatibility, favourable bioavailability, and intrinsic biological activity, making them effective and safe nanocarriers [89]. Limonene also contributed to vesicular deformability, improving drug partitioning while maintaining controlled delivery [84]. Collectively, this architectural balance between hydrophilic and hydrophobic domains ensured efficient solubilization, delayed release, and superior stability. Kinetic modelling provided further insights into the release mechanism. The optimized quatsomes formulation followed Higuchi’s diffusion model, suggesting that drug release was predominantly governed by Fickian diffusion from the vesicular matrix. Overall, the optimized quatsomes provided a strategically advantageous release profile, delivering an initial burst to ensure rapid therapeutic onset, followed by prolonged release to sustain plasma levels. Such a dual-phase behaviour is highly desirable for kidney-targeted therapy, as it not only enhances drug bioavailability but also reduces dosing frequency, improving therapeutic efficacy and patient compliance.

Regarding antioxidant assay, the pronounced effect underscores the ability of quatsomes to potentiate curcumin’s intrinsic antioxidant properties. The superior activity can be attributed to multiple formulation-related advantages, including enhanced solubilization of the poorly water-soluble curcumin, protection against environmental and enzymatic degradation, and facilitated interaction with biological membranes due to the nano-scale size and cationic surface charge of the vesicles [90]. Collectively, these factors likely increased the accessibility of curcumin to reactive oxygen species, thereby accelerating radical scavenging. Importantly, this improvement implies that lower doses of curcumin may achieve comparable or superior therapeutic effects when delivered via quatsomes, potentially reducing systemic toxicity, minimizing cost, and improving compliance.

Moreover, the remarkable stability of the quatsomal formulation can be attributed to a synergistic interplay of multiple factors. The strongly positive zeta potential (+38.30 mV) generated pronounced repulsive electrostatic interactions among vesicles, minimizing the tendency to aggregate [70]. Additionally, cholesterol within the bilayer imparted rigidity and reduced membrane fluidity, thereby enhancing vesicular resilience [81]. The surfactants and terpene further contributed steric stabilization by forming a hydrated interfacial layer that hindered vesicle–vesicle contact [34]. The nanoscale dimensions of the vesicles further influenced a vital role, as their small size promoted uniform dispersion, greater Brownian motion, and increased surface charge exposure, all of which collectively enhanced colloidal stability [34].

Furthermore, the enhanced nephroprotective effect of the optimized quatsomes can be attributed to the combined influence of their physicochemical characteristics and improved delivery behavior. The nanosized vesicular structure contributes to enhanced systemic stability and facilitates efficient renal tissue penetration, allowing better access compared to curcumin suspension. In addition, the positive surface charge may promote electrostatic interactions with renal components, supporting enhanced localization and retention within kidney tissues. Limonene incorporation likely improves membrane fluidity and vesicle deformability, which may enhance cellular uptake and intracellular drug delivery, thereby increasing curcumin availability at the site of action. Regarding hyaluronic acid functionalization, its known affinity for CD44 receptors suggests a possible contribution to cellular uptake, particularly in renal cells where CD44 expression has been reported. However, as no direct mechanistic studies were performed in the current work, CD44-mediated targeting cannot be conclusively confirmed and is therefore considered a potential contributing pathway rather than a definitive mechanism. Overall, these combined features may lead to increased renal accumulation of curcumin. The resulting enhancement in antioxidant and anti-inflammatory activity likely contributes to mitigating cisplatin-induced oxidative stress and tubular injury, as reflected by the observed reduction in serum creatinine and urea levels.

## Limitations of the study

The main limitation of this study is the lack of direct biodistribution analysis to confirm selective renal accumulation of curcumin. The renal targeting effect was inferred from biochemical markers and histopathological findings rather than quantitative tissue distribution studies such as HPLC/LC–MS or fluorescence imaging. Therefore, the proposed targeting mechanisms, including passive renal accumulation and possible CD44-mediated uptake, should be interpreted as supportive hypotheses rather than confirmed pathways. Furthermore, the study did not include a blank nanoparticle (drug-free formulation) group to evaluate the possible intrinsic effect of the nanocarrier system.

## Conclusion

This study demonstrates the successful development of Advanced Quatsomes as a multifunctional, renal-targeted nanocarrier system, strategically engineered to overcome the intrinsic limitations of conventional therapies, including poor renal selectivity and systemic toxicity. Through systematic optimization using a 2^3^ factorial design, an optimized formulation was obtained, characterized by high entrapment efficiency (87.8%), nanoscale particle size (120.55 nm), and a stable cationic surface charge (+38.3 mV), collectively facilitating preferential renal accumulation and enhancing glomerular interaction. Transmission electron microscopy confirmed uniform spherical morphology, while *in vitro* release studies exhibited biphasic Higuchi diffusion kinetics, indicative of controlled and sustained curcumin liberation. Physicochemical stability assessments confirmed preservation of vesicular integrity and functional properties over 3 months. Mechanistically, passive targeting mediated by the nanoscale size and cationic nature of DDAB enhances electrostatic interactions with the glomerular basement membrane and promotes mesangial uptake, while limonene improves vesicle deformability and intracellular penetration. In addition, hyaluronic acid functionalization may contribute to enhanced cellular uptake due to its known affinity for CD44 receptors on mesangial cells. However, as this mechanism was not directly investigated in the present study, CD44-mediated targeting is considered a potential contributing factor to the observed renal uptake and retention rather than a confirmed pathway, which may in turn support improved curcumin delivery and nephroprotective efficacy. Functionally, Quatsomes significantly enhanced curcumin’s antioxidant activity (∼3.8-fold improvement in IC_50_), while *in vivo* evaluation in a cisplatin-induced nephrotoxicity model revealed substantial nephroprotection, evidenced by marked reductions in serum creatinine and BUN levels and preserved renal histology. Collectively, these findings highlight Quatsomes as a rationally designed, mechanistically informed, and clinically promising renal-targeted nanoplatform capable of integrating physicochemical precision, targeted delivery, and therapeutic potentiation, offering a robust and versatile strategy for next-generation renal drug delivery and potential translation into clinical applications.

## Data Availability

The original contributions presented in the study are included in the article/Supplementary Material, further inquiries can be directed to the corresponding authors.
